# Ribosome Fate during Decoding of UGA-Sec Codons

**DOI:** 10.3390/ijms222413204

**Published:** 2021-12-08

**Authors:** Paul R. Copeland, Michael T. Howard

**Affiliations:** 1 Department of Biochemistry and Molecular Biology, Rutgers-Robert Wood Johnson Medical School, Piscataway, NJ 08854, USA; 2 Department of Human Genetics, University of Utah, Salt Lake City, UT 84112, USA

**Keywords:** selenocysteine, selenoprotein, SECIS, recoding, SECIS-binding protein, translation termination, nonsense-mediated decay, ribosome rescue

## Abstract

Decoding of genetic information into polypeptides occurs during translation, generally following the codon assignment rules of the organism’s genetic code. However, recoding signals in certain mRNAs can overwrite the normal rules of translation. An exquisite example of this occurs during translation of selenoprotein mRNAs, wherein UGA codons are reassigned to encode for the 21st proteogenic amino acid, selenocysteine. In this review, we will examine what is known about the mechanisms of UGA recoding and discuss the fate of ribosomes that fail to incorporate selenocysteine.

## 1. Introduction

The genetic code was well established by the mid-1960s and initially thought to be fixed and unalterable. This dogma was challenged in the 1970s and 1980s as a number of studies demonstrated that some mRNAs contain signals in addition to the 64 codons that could modify readout of the genetic code in various ways. The signals may involve intramolecular RNA structures such as hairpins or pseudoknots, combinations of specific nucleotides in tandem, recognition sites for RNA-binding proteins, and inter-molecular base pairing between mRNA and RNA within the ribosome [1]. These signals can induce the ribosome to shift to the −1 or +1 reading frame, skip over bases in the mRNA, or even redefine the meaning of a codon. While many of these mechanisms are quite elegant, the process of redefining a UGA codon from a termination codon to one that encodes selenocysteine (Sec) during translation of selenoprotein mRNAs is arguably one of the most complex. The UGA recoding mechanism involves cis-acting RNA structures, several enzymes required for synthesis of Sec-on-Sec tRNA, a specialized elongation factor, and an impressive list of RNA-binding proteins. Details of Sec synthesis on the Sec tRNA are discussed elsewhere in this issue, and the basic features of the Sec incorporation process have been recently reviewed in detail [2,3]. Here, we present a focused review that delves into the cis and trans-acting recoding signals that promote UGA recoding and the resulting competing outcomes for ribosomes translating UGA-Sec.

## 2. Co-Translational Sec-Incorporation

### 2.1. Cis-Acting Elements

#### 2.1.1. Required Selenocysteine Insertion Sequences

The potential for UGA recoding from termination to Sec incorporation occurs in a subset of organisms from all three domains of life. In all instances examined, an RNA secondary structure called the Sec insertion sequence (SECIS) marks the mRNA for recoding of UGA codons. The product of recoding is the co-translational insertion of Sec and the production of a selenoprotein. Most selenoproteins are oxidoreductases requiring Sec for their function [4,5].

**Figure 1:**
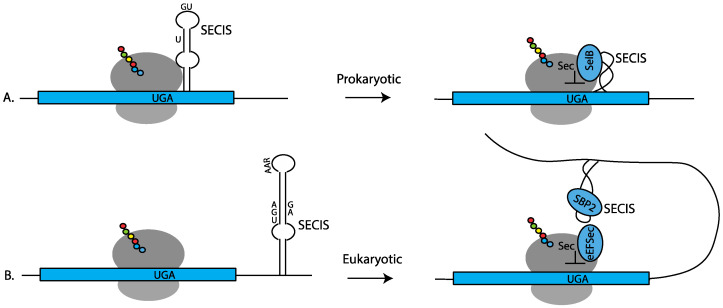
Prokaryotic versus eukaryotic Sec insertion showing the SECIS elements and core components required for Sec-tRNA^Sec^ delivery to the ribosome. (**A**) In prokaryotes, the SECIS element is located just downstream from the UGA codon within the coding sequence. The bulged U and G U nucleotides required for SelB binding are shown. SelB binds to the SECIS element and delivers the Sec-tRNA^Sec^ to the ribosome for UGA recoding. (**B**) In eukaryotes, the SECIS element is located in the 3′ UTR. The SECIS-binding protein, SECISBP2 (SBP2), recruits the Sec-specific elongation factor EEFSEC (eEFSec) to deliver Sec-tRNA^Sec^ to the ribosome for UGA recoding. On the right-hand side of the figure, the SECIS elements are depicted as a helix.

In prokaryotes, the SECIS element is composed of a stem-loop located just downstream from the UGA codon (Figure 1A). The sequence of prokaryotic SECIS elements is highly variable to accommodate the amino acid constraints of the protein being produced. The conserved features are a bulged U in the upper part of the stem-loop and unpaired adjacent G and U nucleotides in the loop (Figure 1A) [6,7]. Sec-incorporation is facilitated by a bifunctional protein, SelB, which consists of SECIS-binding and Sec-tRNA-specific elongation factor domains [8]. The co-location of the SECIS element adjacent to the UGA-Sec codon allows for both positional and temporal delivery of the Sec-tRNA^Sec^ to the ribosome during UGA recoding. Consequently, it came as a surprise when it was shown that SECIS elements in eukaryotes are located in the 3′ UTR of selenoprotein mRNAs [9], necessitating delivery of a signal for UGA recoding from a distance.

One consequence of eukaryotic SECIS action from a distance is that UGA recoding may occur at any in-frame UGA in the message [10,11,12]. Not only does this allow for greater flexibility in the coding region around the UGA codon, but it also allows for recoding of multiple UGA codons during translation of a single mRNA, as is observed in the eukaryotic selenoprotein (SELENOP) mRNA. In eukaryotes, UGA recoding is generally independent of where the UGA occurs in the mRNA; however, as with most phenomena in biology, there are exceptions, and it has been shown that some eukaryotic SECIS elements have evolved the ability to limit Sec-incorporation to UGAs found only in certain regions of the mRNA [13,14].

The conserved features of eukaryotic SECIS elements include an AAR motif in the loop and an AUGA:GA within the stem (Figure 1B) [9,10]. The latter motif is positioned such that the G.A nucleotides form a non-Watson/Crick quartet of sheared G.A/A.G pairs [15,16] that induce a “kink-turn” in the RNA structure. This motif interacts with the RNA-binding protein SECISBP2 (SBP2) to recruit the EEFSEC ternary complex carrying Sec-tRNA^Sec^ (Figure 1B). Although variations in these two motifs exist [4,17,18], these features are sufficient to allow for SECIS search programs to be generated that can predict the entire selenoproteome from genomic sequence alone [4,19]. In addition to facilitating UGA recoding, several studies have demonstrated that some SECIS elements have additional unique molecular functions, such as the ability to direct processive Sec-incorporation at multiple UGA codons in the same transcript [20,21], the ability to alter Sec-incorporation efficiency in response to variations in available selenium [22,23,24], and the ability to discriminate UGA codons depending on their location within the mRNA [13,14]. The remainder of this review will focus on the eukaryotic mechanism of Sec insertion.

#### 2.1.2. Accessory Cis-Acting Selenocysteine Insertion Elements

In cases of stop codon readthrough, where standard near-cognate tRNAs are used to decode the stop codon rather than Sec-tRNA^Sec^, RNA structures and sequences located adjacent to the termination codon have been shown to be stimulatory [25,26,27,28,29,30]. Nucleotides adjacent to UGA-Sec codons were likewise shown to impact UGA-Sec recoding efficiency [31,32,33]. Phylogenetic analyses identified several potential RNA structures downstream from UGA-Sec codons [22,34] that have been designated stop codon/selenocysteine redefinition elements, or SREs. Mutations of the sequences comprising the SRE structure of *selenoprotein N* (*SELENON*) demonstrated the importance of the stem, and of the sequences within the loop and the spacer separating it from the UGA-Sec codon to inhibit termination and stimulate Sec-incorporation [34,35,36]. The signals for two of these structures in SELENON and *selenoprotein T* (*SELENOT*) were also independently identified in a genome-wide search for deeply conserved RNA structures [37]. In addition to the SREs, structural and sequence motifs located close to a UGA-Sec codon in selenoprotein S (*SELENOS*) mRNA have been shown to impact Sec-incorporation. The sequence motif referred to as the *SELENOS* positive UGA recoding element, or SPUR, has been shown to enhance Sec-incorporation efficiency [38,39]. The authors propose that the SPUR element interacts with the SECIS element itself to facilitate Sec incorporation.

### 2.2. Trans-Acting Factors

#### 2.2.1. The Core SECIS-Binding Proteins

Just over 20 years ago, two eukaryotic trans-acting factors essential for Sec-incorporation were identified: the SECIS RNA-binding protein (SECISBP2), and the Sec-tRNA^Sec^ specific elongation factor (EEFSEC) [40,41,42]. Unlike the bacterial SelB, SECISBP2 lacks the ability to bind the Sec-tRNA^Sec^. This function is provided by EEFSEC.

EEFSEC differs from the standard eukaryotic elongation factor (EEF1A) in that it has the ability to interact with SECISBP2, has a higher affinity for GTP than GDP [41,43], and has an extended C-terminal extension that, unlike EEF1A and EF-Tu, undergoes a conformational change upon guanine nucleotide exchange [44]. The latter observation suggests a non-canonical mechanism for release of Sec-tRNA^Sec^ to the ribosome.

SECISBP2 contains a canonical L7Ae RNA-binding domain [45,46,47] that is responsible for interactions with the kink-turn motif of the SECIS element, a central Sec-incorporation domain (SID) that is required for Sec-incorporation but not SECIS binding, and a poorly conserved N-terminal domain of unknown function. A crucial insight came from the observation that SECISBP2 and EEFSEC interact in a manner that is stimulated by the presence of Sec-tRNA^Sec^ [48], suggesting a mechanism whereby SECISBP2 would only recruit EEFSEC carrying Sec-tRNA^Sec^ and would dissociate from EEFSEC upon delivery of Sec-tRNA^Sec^ to the ribosome.

While SECISBP2 is clearly a central player in Sec-incorporation, studies of SECISBP2 deletions in mice [49] and cultured mammalian cells [50] revealed, surprisingly, that SECISBP2 is not absolutely required for Sec-incorporation, although the efficiency of UGA recoding is greatly reduced in its absence. One possible explanation is that SECISBP2L, a paralogue of SECISBP2, may compensate for SECISBP2 in its absence or under certain physiological conditions [51]. An alternative explanation involves direct binding of EEFSEC to the SECIS element. Initial studies of SECISBP2- and EEFSEC-binding to SECIS RNA in vitro illustrated direct binding of EEFSEC in the absence of SECISBP2 [42]. SECISBP2 may not be absolutely required, but rather strongly enhances efficient EEFSEC ternary complex delivery to the ribosome or facilitates exchange following UGA-Sec recoding, perhaps through direct interactions with the 28S ribosomal RNA [52,53].

Emerging from these early studies is a picture in which the SECIS elements act as a scaffold for RNA-binding proteins and this ribonucleoprotein complex recruits EEFSEC to accommodate UGA recoding by Sec-tRNA^Sec^. In the following section, we will discuss additional SECIS-binding proteins that have been proposed as part of the SECIS ribonucleoprotein complex, and how these may further modulate the efficiency of UGA-Sec recoding.

#### 2.2.2. Accessory Trans-Acting Factors

Levels of complexity were added to the mechanism of Sec-incorporation with the discovery of several additional SECIS-binding proteins. The first of these, nucleolin (NCL), best known for its role in ribosome biogenesis, was shown to bind specifically to the SECIS element [54] around the same time as the discovery of SECISBP2. Subsequent studies showed that NCL binds to the upper part of the SECIS stem for a subset of selenoprotein mRNAs, and thus is unlikely to compete with SECISBP2-binding [55]. Knockdowns of NCL cause a decrease in levels of selenoproteins without changing mRNA levels or localization, suggesting that it is a positive regulator of selenoprotein translation.

In contrast to NCL, the SECIS-binding ribosomal protein L30 contains an L7Ae domain and competes directly with SECISBP2-binding at the kink-turn motif of SECIS elements [56]. The canonical role of L30 includes interaction with the 60S rRNA subunit [57] mediated through binding to a kink-turn motif in helix 58 of the 28S rRNA, as well as binding the 5′ UTR of its own mRNA to auto-regulate expression [58]. Increasing levels of L30 in cultured cells were shown to enhance Sec-incorporation [59], whereas addition of free L30 to in vitro translation reaction was shown to decrease UGA-Sec recoding [56]. A possible explanation for these disparate results comes from the finding that ribosome-associated L30 has a higher affinity for the SECIS element than free protein. One model proposes that within the context of the ribosome, L30 may have transient interactions with the SECIS element that displace SECISBP2 and stabilize a SECIS conformation that is required for EEFSEC delivery.

Several years after the identification of L30 as a SECIS-binding protein, EIF4A3, a member of the DEAD-box family of RNA-dependent ATPases, was also shown to bind SECIS elements contained in selenoprotein mRNAs that are known to be sensitive to degradation and have reduced UGA recoding efficiency when selenium is limiting [60]. Mapping of the EIF4A3-binding site demonstrated that it overlaps with the SECISBP2-binding site, and it was further shown that when selenium is limiting, there is an increase in cellular EIF4A3 protein expression. Importantly, EIF4A3 is also known to be a key component of the exon junction complex (EJC) involved in nonsense-mediated decay (NMD) [61,62,63], suggesting a possible direct link between inhibition of Sec-incorporation and degradation of selenoprotein mRNAs.

#### 2.2.3. Selenoprotein mRNA 5′ Cap Modifications and Recruitment of the SMN Complex

Most mRNAs contain a 5′ m7G cap that plays important roles in RNA processing and stability and is bound by the translation initiation factor EIF4E as a key step in the process of translation initiation [64]. Several of the selenoprotein mRNAs are inefficiently recognized by EIF4E because the cap is hypermethylated by the trimethyl-guanosine synthase (TGS1) via a pathway related to that which processes small nuclear RNAs and snoRNAs [65,66]. The tri-methyl guanosine (TMG) capped selenoprotein mRNAs are localized to the cytoplasm and actively associate with ribosomes; at least one selenoprotein mRNA, GPX1, appears to require TMG to support efficient translation.

TGS1 is recruited to selenoprotein mRNAs by interactions between SECISBP2 and the survival of motor neuron protein complex (SMN). The SMN protein is a component of a ribonucleoprotein assembly chaperone pathway first described as being essential for assembly of small nuclear RNPs involved in splicing [67]. In addition to the selective 5′ TMG modification of selenoprotein mRNAs, perhaps the recruitment of the SMN complex to select selenoprotein mRNAs helps chaperone the formation of functional ribonucleoprotein complexes involved in UGA recoding.

Most recently, the RNA-binding protein PTBP1 has been shown by RNA affinity chromatography to interact with 3′ UTR sequences of SELENOP in the U-rich region separating the two 3′ UTR SECIS elements [68]. Deletion of this region inhibited the regulation of translation that normally occurs during oxidative stress in cultured human liver cells, indicating that there may yet be undiscovered regulatory elements in the 3′ UTRs of individual selenoproteins.

Although many of the key components of the Sec-incorporation machinery have been identified, we have an incomplete picture of the dynamic nature of the Sec ribonucleoprotein complex, how it delivers EEFSEC and the Sec-tRNA^Sec^ to the ribosome during UGA recoding, and how this process competes with the standard decoding process of translation termination. It has been clearly established through in vitro reporter assays and in vivo ribosome profiling experiments that UGA recoding is an inherently inefficient process (with the notable exception of Sec-incorporation at the C-terminal UGA-Sec codons of SELENOP mRNAs), such that the majority of ribosomes that initiate translation on selenoprotein mRNAs fail to incorporate Sec and never reach the natural termination codon.

**Figure 2:**
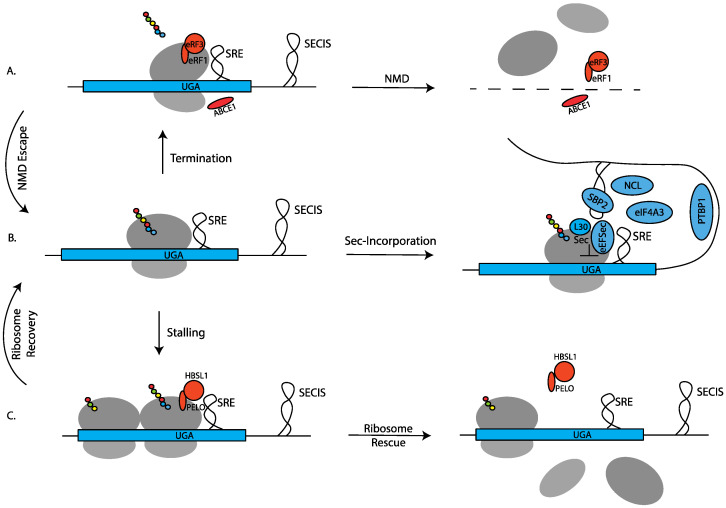
Possible fates of ribosomes encountering UGA-Sec codons. (**A**) Ribosomes failing to incorporate Sec may prematurely terminate translation, leading to nonsense-mediated decay (NMD) or NMD escape and continued translation by ribosomes located upstream of the UGA codon. (**B**) Sec-incorporation mediated by Sec insertion machinery. (**C**) Ribosomes that fail to incorporate Sec or terminate may be subject to various translational quality control pathways leading to ribosome release or recovery.

Several outcomes can be envisioned for ribosomes that fail to incorporate Sec: (1) ribosomes may decode the UGA-Sec codon as a stop codon and terminate translation (Figure 2A, and in some cases this event will be recognized as premature termination leading to mRNA degradation through the nonsense-mediated decay (NMD) pathway; (2) ribosomes may stall at or near the UGA-Sec codon. Stalled ribosomes may either be removed by ribosome rescue or be rescued and continue translation (Figure 2C). RNAs that escape NMD or those on which ribosomes have been rescued may resume translation and go on to incorporate Sec (Figure 2B). What is known about these competing events will be discussed in Section 3.

## 3. Competing Ribosome Fates at UGA-Sec Codons

Every step of the information-flow pathway in a living cell is monitored and kept accurate by quality control mechanisms. Replication has DNA repair, transcription has proofreading Pol II, tRNA aminoacylation has noncognate hydrolysis, and ribosomes both execute and are subjected to multiple control mechanisms during translation. These quality control pathways are broad and arguably nonspecific, so naturally there are a multitude of exceptions that must either evade or modify specific components of quality control. As a case in point, the mechanism by which mRNAs containing a misplaced in-frame stop (also known as nonsense) codon are sensed and shunted to a degradation pathway was discovered in 1979 [69], and a vast literature provides substantial mechanistic insight into this process termed nonsense-mediated decay [70]. The prevailing model posits that a stop codon is considered to be in the “wrong” position if it is not in the last exon. Thus, if the ribosome encounters a premature stop codon, it will “sense” the existence of a downstream exon/exon boundary, which is bound by a host of factors collectively referred to as the exon junction complex (EJC). Essentially the EJC is thought to cause inefficient translation termination which is sufficient to recruit the lynchpin NMD factor UPF1, which in turn recruits other factors required to initiate mRNA decay. It is important to stress that inefficient termination is sufficient to induce NMD, thus explaining the considerable evidence that EJC-independent NMD does occur and represents a significant fraction of total NMD events, particularly in organisms that have very few introns (e.g., *Saccharomyces cerevisiae*).

Selenoprotein mRNAs, with in-frame stop codons that are reprogrammed to allow Sec incorporation, stand as interesting case studies in the regulation of NMD. When selenium is replete, it is plausible to assume that efficient use of UGA as a sense codon would be sufficient to prevent NMD activation. However, it has long been reported that the efficiency of Sec incorporation is low, in the order of 10–25% [20,32,35,71]. Since the vast majority of Sec codons lie upstream from exon/exon boundaries [72], nearly every case of failed Sec incorporation might initiate NMD. Although the half-lives of all selenoprotein mRNAs have not been directly measured under the array of conditions required to precisely answer this question, the reality is that steady state levels of selenoprotein mRNAs are sufficient to provide adequate selenoprotein production under normal conditions. This may be due to the fact that selenoprotein mRNAs are shielded from NMD factors during incorporation, so the termination events that do occur are not able to signal the NMD machinery. This hypothesis was generally supported by a study where liver-specific knockout of SECISBP2 or tRNA^Sec^ resulted in an overall 60–70% reduction of selenoprotein mRNA levels [49]. However, a deeper analysis of the relative contributions of translation efficiency and mRNA abundance revealed a complex array of differential regulation, depending on the identity of the selenoprotein mRNA. For example, their findings for GPX1 were consistent with prior work that showed it to be a strong NMD target during selenium deficiency. This stands in contrast to GPX4, whose mRNA levels are dependent on SECISBP2, but not on selenium or Sec-tRNA^Sec^ levels. In addition, there appeared a third case, SELENOT, where mRNA levels were reduced but the translational efficiency remained the same. Part of this complexity is undoubtedly due to the existence of an SECISBP2 orthologue, SECISBP2L, which is very likely responsible for supporting selenoprotein production when SECISBP2 is absent. Overall, it is clear that active Sec incorporation is essential for maintaining selenoprotein mRNA levels, albeit to varying extents, but the question remains as to how much higher they would be if there were no in-frame UGA codon. One study did tangentially address this question by stably transfecting cDNAs encoding the zebrafish and human versions of SELENOP into the human hepatocyte cell line HepG2. One version of the cDNA was wild-type, which contains multiple Sec codons, and the other had replaced those with Cys codons. Quantitative RT-PCR revealed that the steady state mRNA level for the Cys codon-containing construct was about twice that of the Sec codon counterpart [73]. Interpreting this result is confounded by the fact that SELENOP is an exception among selenoproteins in possessing multiple UGA codons. Overall, while there is no doubt that NMD and Sec incorporation are deeply intertwined, there are still mechanistic questions that remain unanswered.

As shown in Figure 2, a byproduct of inefficient Sec incorporation is stalled ribosomes. In general, ribosome-stalling is an undesirable event that can result from a multitude of aberrant or regulated translation elongation reactions, so a surveillance and quality control pathway termed no-go decay (NGD) evolved to prevent ribosomes from accumulating on inefficiently translated mRNAs [74,75]. As such, the components that make up the NGD pathway may also play a role in regulating the fate of selenoprotein mRNAs. Perhaps not surprisingly, the two key factors in signaling NGD (PELO and HBS1) are evolutionarily related to the termination factors eRF1 and eRF3. One of the key signals for the NGD pathway event is an empty ribosomal A site, which would occur during inefficient translation elongation. Similar to a translation termination reaction, where an eRF1/eRF3 complex recognizes the ribosomal A site at stop codons, the PELO/HBS1 complex accesses any A site that is not occupied by an elongation complex and recruits factors that release the ribosome and degrade the mRNA. So, in the case of a Sec codon, three different complexes are competing for the same site: EEFSec/Sec-tRNA^Sec^, eRF1/eRF3, and PELO/HBS1. Although some attention has been paid to the mechanism of the interplay between termination and Sec incorporation [31,32], very little has been studied about the role of the NGD pathway as a potential regulator. It is likely, therefore, that Sec incorporation efficiency is directly related to the ability of Sec-tRNA^Sec^ to outcompete the access of eRF1 or PELO. The multitude of factors that would impact this competition include local RNA structure, codon sequence context, relative concentrations of the A-site binders, and the nascent peptide chain sequence that is known to regulate the processivity of ribosome transit during translation elongation [76]. The major mechanistic question for the field centers around the extent to which Sec incorporation “actively” competes with these processes. For example, specific occlusion of PELO or eRF1 by the SECISBP2/SECIS complex, even in the absence of an EEFSec/Sec-tRNA^Sec^ complex, would represent an active competition. On the other hand, termination and NGD might be passively out-competed purely as a function of the relative concentrations of factors. Intriguingly, it has been reported that increasing the amount of eRF1 (but not eRF3) caused an increase rather than the expected decrease in Sec incorporation in transfected cells [31]. Similarly, the addition of eRF1 to an in vitro translation system also failed to inhibit Sec incorporation [32]. Taken at face value, these results may favor the “active” model of Sec incorporation where specific events prevent termination regardless of eRF1 concentration. While it is likely that a combination of active and passive processes is at play, a thorough investigation of the role that NGD factors might play in regulating the efficiency of Sec incorporation will be required to shed light on any role that PELO (or PELO exclusion) may play.

In the context of considerable complexity regarding processes that monitor A-site occupancy during the translation elongation reaction, another quality control mechanism monitors the stalling of ribosomes. The ribosome quality control (RQC) system induces a general cellular stress pathway that is coordinated by activation of the heatshock protein Hsf1 when nascent chain peptides are stalled [77]. The outcome of activating this pathway is ribosomal subunit ubiquitination and nascent chain degradation [78]. More recent work has shown that the signal is actually conveyed by “colliding” ribosomes [79]. Again, since ribosomes stall on many selenoprotein mRNAs, the interplay between the RQC pathway and Sec incorporation stands as yet another potential contributor to the overall efficiency. Indeed, the question of whether ribosome-stalling on selenoprotein mRNAs is sufficient to signal the stress pathway raises an intriguing possibility. While it is unlikely to be the case under normal conditions, the RQC pathway could, however, be an important signaling mechanism for the cell to detect limiting selenium concentrations, because selenoprotein mRNAs that are not turned over by NMD would likely accumulate collided ribosomes.

## 4. Conclusions

In this focused review, we have highlighted the fact that Sec incorporation must coexist with multiple quality control pathways. In theory, each of these pathways should effectively reduce Sec incorporation, but the Sec incorporation machinery co-evolved to deploy mechanisms that both subvert and exploit these pathways to optimize efficiency and signal stress conditions. The key for future exploration will be to decipher the mechanistic bases of the workarounds that organisms were required to evolve in order to effectively utilize Sec.

## Data Availability

Not applicable.
